# Radial Artery Access for Hepatic Chemosaturation: The First Description of Technical Feasibility

**DOI:** 10.7759/cureus.18852

**Published:** 2021-10-18

**Authors:** Joshua P Frost, Pavan Najran, Jon Bell, Damian Mullan

**Affiliations:** 1 Radiology and Interventional Radiology, The Christie NHS Foundation Trust, Manchester, GBR

**Keywords:** liver cancer directed therapies, interventional radiology guided embolization, liver metastases, hepatic chemosaturation, radial artery access

## Abstract

Chemosaturation with percutaneous hepatic perfusion (CS-PHP; Hepatic CHEMOSAT® Delivery System, Delcath Systems Inc, Wilmington, Delaware) is an interventional radiology procedure that delivers high doses of melphalan, a chemotherapeutic agent, directly to the liver in patients with unresectable primary and secondary liver tumours.

Traditionally, CS-PHP is delivered by arterial access via the femoral artery. However, there can be many risks and adverse effects associated with femoral artery punctures, such as retroperitoneal haemorrhage and haematoma formation. The monitoring and bed rest required following the removal of a femoral arterial catheter may also cause significant distress to patients as they remain immobile, potentially prolonging their stay in hospital. The radial artery is an alternative access point, with fewer reported adverse events and increased patient tolerance when compared with femoral access.

This case report details the first reported use of Hepatic CHEMOSAT® therapy being delivered via the radial artery. Two patients received hepatic chemosaturation with no reported complications. This report demonstrates that access via the radial artery is a feasible alternative for the delivery of chemotherapy, which may reduce morbidity and the risks usually associated with femoral access.

## Introduction

Hepatic chemosaturation

Chemosaturation with percutaneous hepatic perfusion (CS-PHP; Hepatic CHEMOSAT® Delivery System; Delcath Systems Inc, Wilmington, Delaware) is an interventional radiology procedure that delivers high doses of melphalan directly to the liver in patients with unresectable primary and secondary liver tumours [1]. The most common primary malignancy treated is ocular melanoma, though reports of treatment for colorectal cancer, cutaneous melanoma and breast cancer also exist [2]. Hepatic response rates by Response Evaluation Criteria in Solid Tumors (RECIST) criteria (assessment of tumour shrinkage) tend to vary, with studies quoting figures from 58-75% [3], though response rates to uveal melanoma alone have been reported up to 83% [4]. 

In CS-PHP, melphalan is delivered directly into the hepatic artery via a catheter inserted from the femoral artery into the proper hepatic artery. A double-balloon catheter, advanced through the right femoral vein, is positioned in the inferior vena cava (IVC) with the cranial balloon in the right atrium-IVC junction and the caudal balloon in the infrahepatic IVC above the renal veins. This allows hepatic inflow to be isolated, preventing direct hepatic venous outflow and limiting systemic melphalan exposure [3]. An extracorporeal circuit is then established with blood from the isolated hepatic system entering a veno-veno bypass pump prior to undergoing chemofiltration, where the blood is then returned to the body via a catheter inserted into the internal jugular vein. There can be pronounced and sudden variations in blood pressure during isolation of the liver, requiring general anaesthesia and vasopressors, which can cause generalised arterial contraction. Fluid input and output is therefore closely monitored, with the patient catheterised to record urine output.

During the procedure, high-dose heparin (350 units/kg) is administered to maintain activated clotting time at therapeutic levels for haemofiltration (activated clotted time > 450s). Following the procedure, anticoagulation is reversed and vasopressor support is withdrawn. The vascular access sheaths are removed when the patient's coagulation profile is normal. A vascular occlusion device can be used to seal a femoral artery puncture, but direct pressure is frequently used as an alternative. Extubation and recovery from general anaesthesia is traditionally performed in the radiology theatre environment. When conscious, patients are transferred to a high dependency unit (HDU) where they are monitored for six to 16 hours before being moved onto a general ward for up to three days prior to discharge [3].

Complications of using femoral access

Traditionally, CS-PHP is delivered with arterial access via the femoral artery. However, there are risks and adverse effects associated with femoral artery puncture. Retroperitoneal haemorrhage (RPH) from femoral artery access is a well-documented, potentially fatal complication of transfemoral catheterisation. The reported incidence of RPH caused by transfemoral catheterisation procedures ranges from 0.15-6%, with reported mortality rates of 4-12% [5]. Furthermore, to reduce the risk of vascular complications, post-femoral puncture care relies mainly on bed rest, with patients having to remain supine for up to four hours post-catheter removal [6]. The malaise caused by prolonged supine positioning and immobility may be compounded by difficulty urinating, pelvic discomfort, and anxiety - factors that may also be predictors for a vasovagal response during sheath withdrawal [6].

Complications of using a vascular occlusion device

Vascular occlusion devices commonly used to facilitate haemostasis at the puncture site are also associated with their own potentially serious complications, including incorrect deployment, device entrapment, lower limb ischaemia/arterial stenosis, or infection. There is therefore an increased risk of patients requiring urgent vascular surgery for these arterial complications when compared with manual compression alone [7-9].

Transradial arterial access

The radial artery is an alternative access point, with fewer reported complications when compared with femoral artery access [10-12]. In coronary artery angiography, the European Society of Cardiology recommends transradial access as the first-line approach for coronary diagnosis and therapy because this method is associated with a lower haemorrhage risk, a shorter length of hospital stay, a shorter period of immobilization, lower costs, and greater levels of patient satisfaction than with transfemoral access [11]. This has lead the technique to become a more acceptable and often preferred access point in interventional radiology.

Despite this, transradial access is not without its own potential complications, with operators, in particular, expressing concerns with the risk of radial artery occlusion (RAO). However, a study by Hadjivassiliou et al. [13] showed that from 593 procedures performed using distal transradial artery access, only seven small haematomas were observed as a complication, with no reported cases of RAO. This demonstrates that when an appropriate haemostasis protocol is followed, the risks of a severe adverse event following transradial access can be minimised greatly. Additionally, access site complications are particularly relevant in the settings of anticoagulation and thrombocytopenia. Transradial access has been shown to be a safe and feasible option for both thrombocytopenic and anticoagulated patients [14].

Furthermore, Thakor et al. [10] found that 98% of interventional radiology patients who underwent a radial access procedure following a previous femoral access procedure would preferentially choose radial access for any subsequent procedures. Radial artery access has subsequently been performed routinely in preference to femoral access for angiographic procedures at our institution since 2015.

With these points in consideration, this case report highlights two cases where transradial access was used to deliver CS-PHP. To our knowledge, this is the first reported use of the radial artery for the delivery of hepatic chemosaturation.

## Case presentation

**Figure 1:**
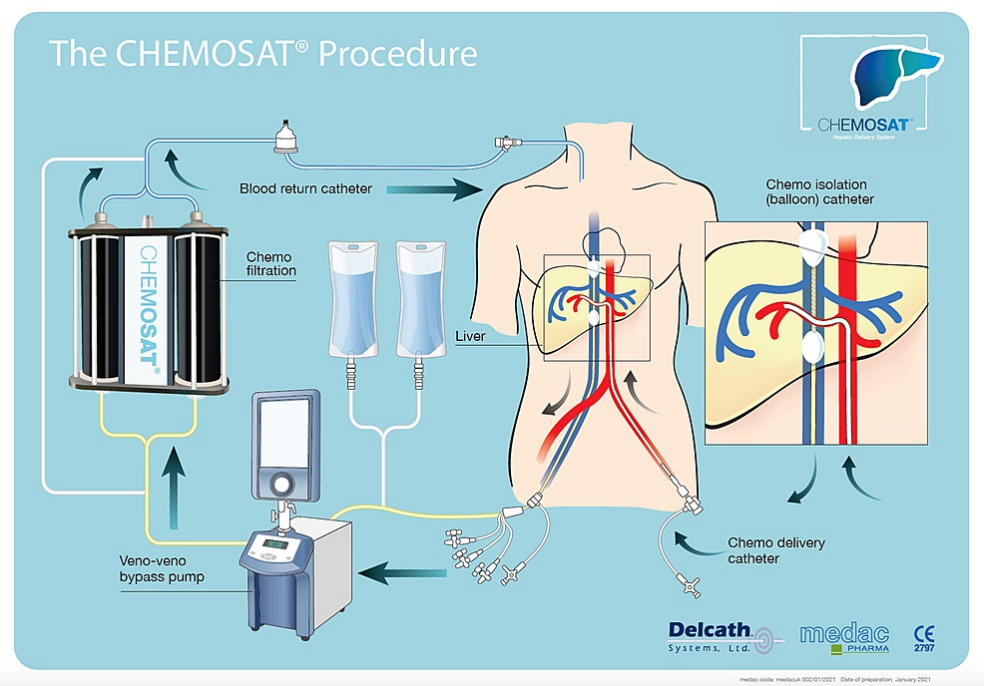
Overview of CS-PHP treatment Reproduced with permission from Delcath Systems Inc. CS-PHP: chemosaturation with percutaneous hepatic perfusion

**Figure 2:**
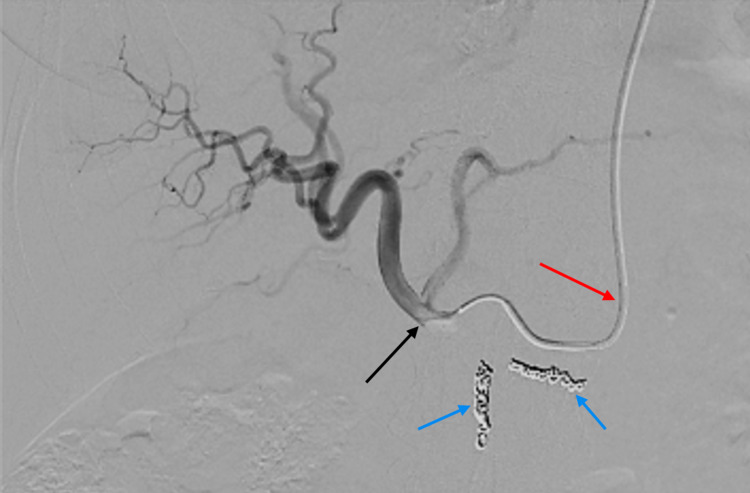
Fluoroscopic image showing contrast injection through the radial catheter into the proper hepatic artery The catheter (red arrow), having been inserted via the left radial artery, has been advanced down through the thoracic aorta and coeliac axis to the proper hepatic artery (black arrow). Coils have been placed in the gastroduodenal artery and right gastric artery to prevent reflux of melphalan (blue arrows).

**Figure 3:**
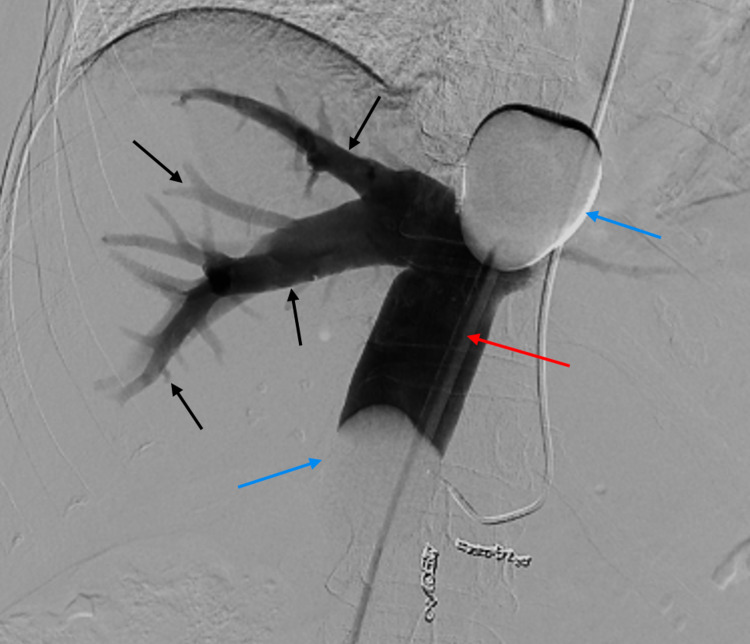
Fluoroscopic image (digital subtraction angiography) showing contrast injection through the venous catheter Contrast delivered via the venous catheter (red arrow) outlines the hepatic venous system (black arrows). The balloons (blue arrows) sit in the right atrium superiorly and above the renal veins inferiorly, isolating the hepatic venous system.

**Figure 4:**
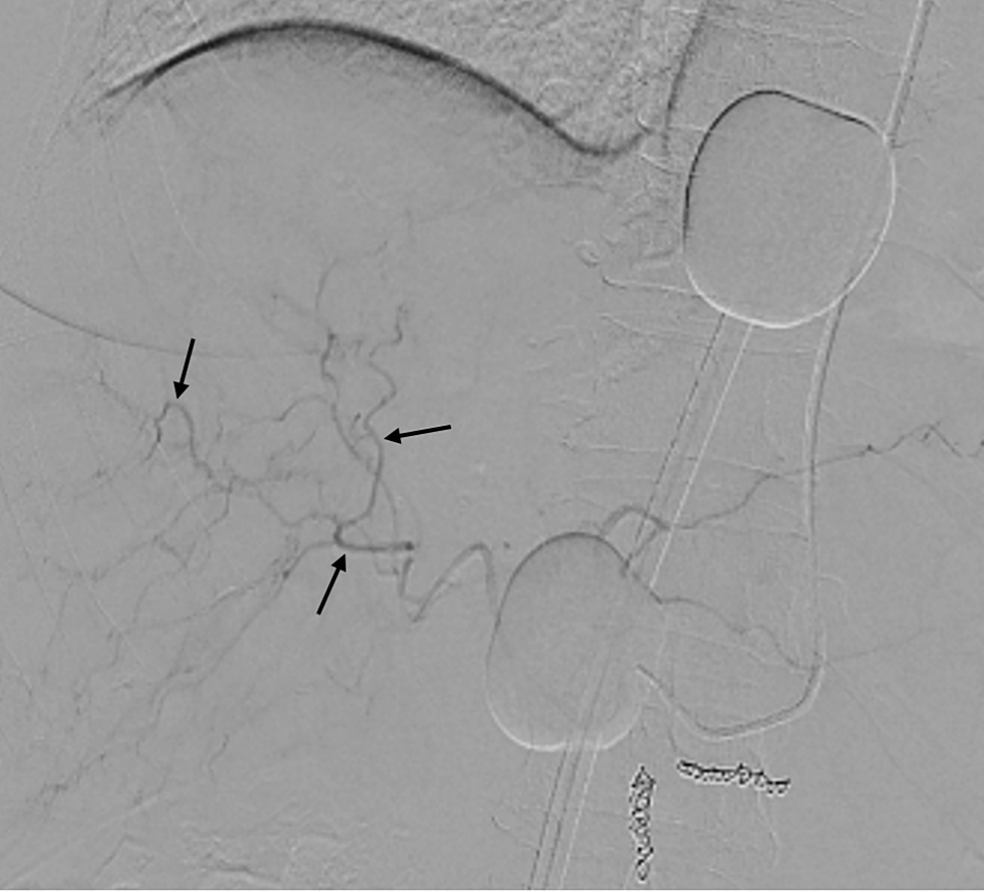
Fluoroscopic image with contrast injection (during procedure) Contrast injection into the hepatic arterial vasculature demonstrating vasospasm of the hepatic arterial vessels (black arrows). This is caused by vasopressor support given to the patient whilst undergoing haemofiltration.

**Figure 5:**
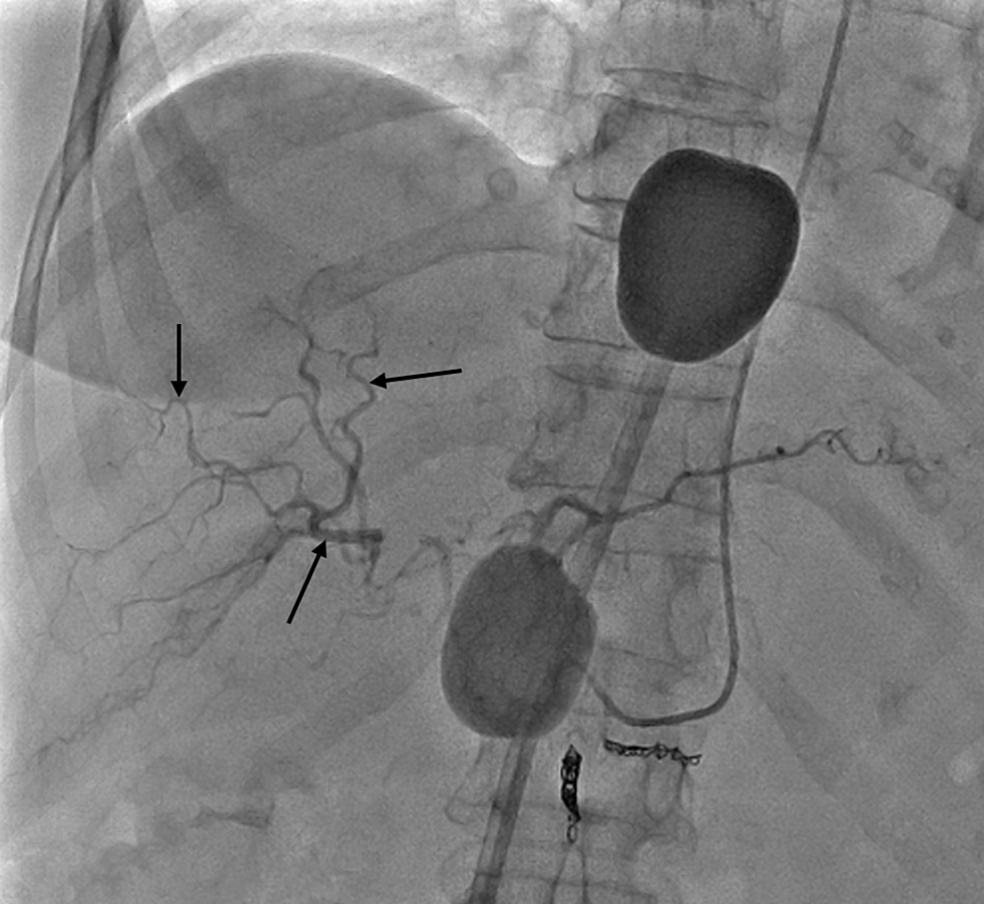
Fluoroscopic image with contrast injection (post-procedure) Contrast injection showing hepatic arterial vasculature returning to its pre-vasospastic state following withdrawal of vasopressor support post-haemofiltration.

Aside from the delivery of chemotherapy via a transradial arterial catheter, the following cases followed the same procedural setup for CS-PHP as detailed in the schematic overview (Figure 1). Navigation of a transradial arterial catheter into the proper hepatic artery was performed without complication in both cases (Figure 2). A double-balloon catheter inserted via the right femoral vein was then used to isolate the hepatic venous system to limit systemic chemotherapy exposure (Figure 3). Inflation of the double-balloon catheter and commencement of haemofiltration precipitated a large drop in blood pressure that necessitated the use of vasopressor support, with which the effects on the hepatic arterial vasculature can be seen in Figure 4. Following the withdrawal of vasopressor support post-haemofiltration, the hepatic arterial vasculature returned to its pre-vasospastic state (Figure 5).

Case 1

**Figure 6:**
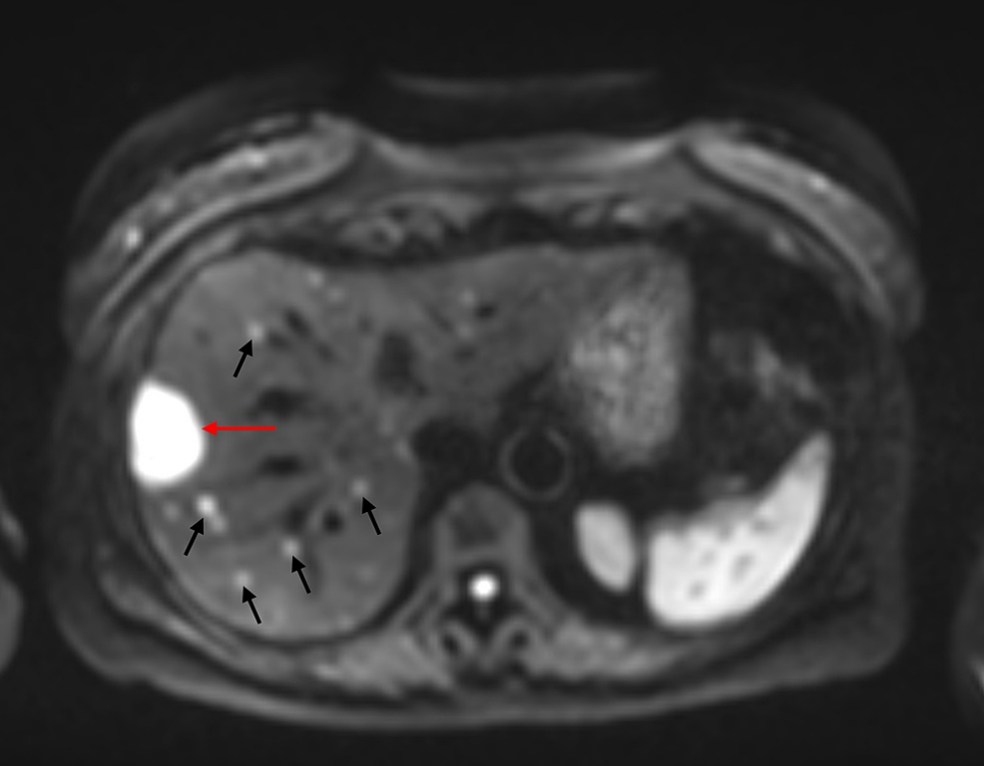
MRI liver with hepatobiliary contrast Diffusion-weighted imaging of the liver showing dominant metastasis in segment 8 measuring 3.6 cm (red arrow) and multisegmental subcentimeter metastases (black arrows).

A 75-year-old woman was referred to our hospital with uveal melanoma of the ciliary body and choroid. The patient had a past medical history of rheumatoid arthritis and hypertension. The patient had previously undergone treatment, with enucleation of the right eye and four cycles of systemic anti-cancer therapy (SACT) consisting of ipilimumab and nivolumab. However, a follow-up MRI scan demonstrated around 10-15 metastases within the liver (Figure 6). The patient was referred to MDT where it was decided they were a suitable candidate for CS-PHP.

At the time of the report, the patient had undergone three cycles of CS-PHP. The first was performed with access via the femoral artery at a different institution, whereas the following cycles were performed with access via the distal left radial artery.

Ultrasound-guided puncture of the 2.3 mm left distal radial artery was performed, with a 5 French radial catheter (Performa Ultimate™; Merit Medical Systems, Inc., South Jordan, UT) to access the coeliac trunk, and a 2.4 French Microcatheter (PROGREAT® Microcatheters; Terumo Interventional Systems, Somerset, NJ) to access the treatment position in the common hepatic artery (Figure 2). The procedure was performed without complications, and the left radial sheath was removed after heparin reversal with protamine sulfate. Removal was performed without complication on both occasions, using a radial artery compression device (PreludeSYNC™ Radial Compression Device; Merit Medical Systems, Inc.) to provide 'hands-free' haemostasis. Both the right jugular sheath and right femoral sheath were removed without complications on the ward following correction of international normalized ratio (INR).

Case 2

A 55-year-old woman was initially referred to our hospital with metastatic breast cancer with a left-sided primary, grade 2, mixed ductal/lobular invasive carcinoma, ER8, HER-2 1+. CT staging showed liver metastases. The patient completed four cycles of FEC (fluorouracil, epirubicin hydrochloride, and cyclophosphamide)/four cycles of docetaxel. The patient went on to have a left mastectomy and axillary node clearance and was started on tamoxifen whilst receiving adjuvant radiotherapy to the chest wall post-mastectomy.

**Figure 7:**
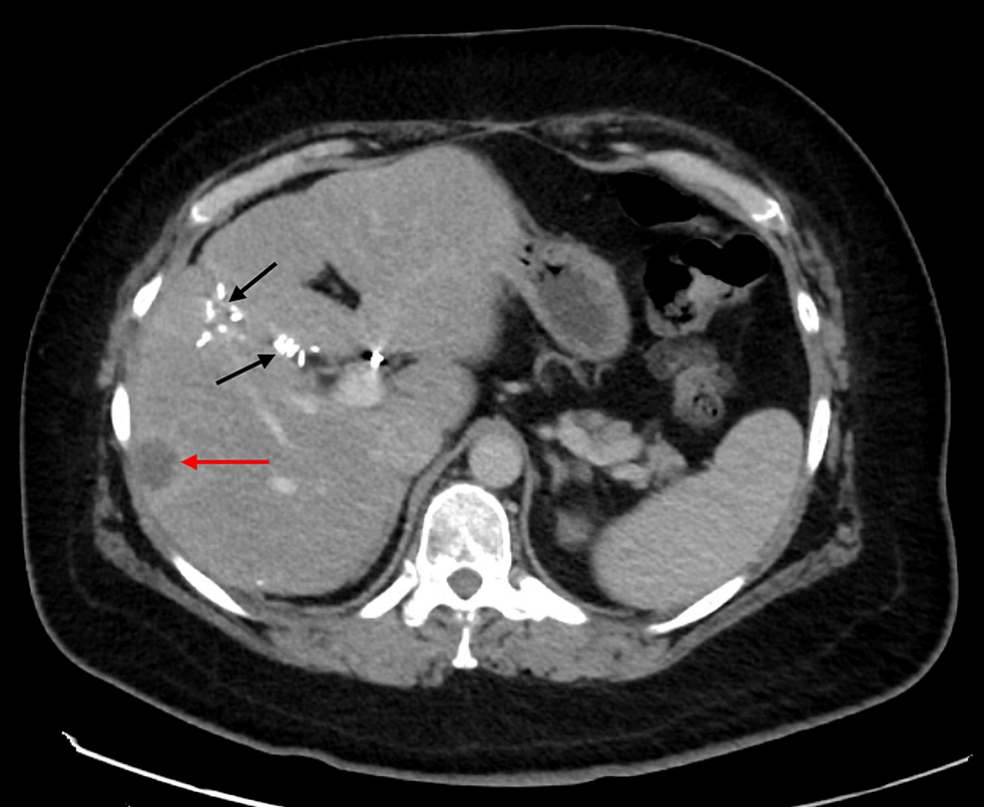
CT abdomen/pelvis in the portal venous phase Scan showing dominant liver metastasis of ~20 mm (red arrow). Surgical clips are seen in situ (black arrows) due to previous surgical resection of liver metastases.

CS-PHP was then considered at a tumour board meeting as an option for residual liver metastases. The patient underwent two cycles of CS-PHP with the resolution of all but one metastasis. Following this, the patient underwent a segmentectomy of the only remaining visible metastasis in the liver. After a period of remission, further scans revealed new, inoperable liver metastases, prompting recommendation for a further cycle of CS-PHP (Figure 7). On this occasion, the 2.1 mm right distal radial artery was used as the access site, as the left radial artery was 1.7 mm and deemed too small to catheterise. Negotiation into the hepatic arterial system was achieved with a 5 French radial catheter (Performa Ultimate™; Merit Medical Systems, Inc.) and a 2.4 French Mircocatheter (PROGREAT® Microcatheters; Terumo Interventional Systems) and the position was confirmed fluoroscopically. The right radial access sheath was removed after heparin reversal with protamine sulfate. This was performed in the radiology theatre without any significant bleeding. A compression band (PreludeSYNC™ Radial Compression Device; Merit Medical Systems, Inc.) was placed to provide 'hands-free' haemostasis. Both the right jugular and femoral vein sheaths were removed without complication on ITU following normalisation of the patient's clotting profile.

## Discussion

Worldwide, CS-PHP has traditionally been performed with access via the femoral artery [2-3]. To our knowledge, the cases presented are the first reported uses of the radial artery for hepatic chemosaturation.

When we consider the risks and morbidity associated with femoral artery puncture, these cases have demonstrated that access via the radial artery is a safe and feasible alternative to deliver melphalan into the proper hepatic artery. There were no reported complications associated with either insertion of the catheter or removal, with compression alone used to encourage haemostasis.

The advantages of this technique are inherent, as patients are not required to remain supine following removal of the arterial sheath, allowing HDU nursing care to be delivered in a semi-upright position in a bed. The patient can mobilise sooner following the procedure, reducing discomfort and anxiety. Whilst CS-PHP patients are catheterised, radial artery access can allow earlier mobilisation for other aspects of self-caring or comfort in comparison to femoral artery access. In both cases, removal of the femoral and jugular venous sheaths was uncomplicated and did not require significant monitoring or bed rest.

A limitation of this study was the small sample size, with two cases reported. However, in a procedure where traditionally transfemoral access is the norm, these cases demonstrate that transradial arterial access is a viable alternative to transfemoral access, especially when we consider that radial artery access has been shown to be superior to femoral artery access in reducing the incidence of haemorrhagic events [12]. Whilst the large dose of heparin given during CS-PHP is reversed prior to both femoral and radial sheath removal, it is perhaps debatable whether radial artery access alone would decrease the relative risk of haemorrhage in a technically proficient ultrasound-guided puncture of the femoral artery. Removal of the radial sheath was immediate following heparin reversal and was performed in the radiology theatre room in both cases, reducing the anxiety of removing a femoral arterial sheath in an HDU setting. The compression was 'hands-free,' with a compression band, allowing clinical care to be concentrated in other areas.

Radial artery access may also offset the rare but documented risks that can be associated with vascular closure devices, such as acute occlusion and infection [9], and may provide a cost-saving benefit by not requiring their use.

## Conclusions

This case report details the first reported use of Hepatic CHEMOSAT® therapy being delivered via the radial artery, where traditionally it has been delivered via the femoral artery. In both patients described, access via the radial artery/distal radial artery was reported, with no complications. When we consider the risks and morbidity that can be associated with femoral artery puncture, the radial artery is a feasible alternative for the delivery of Hepatic CHEMOSAT® therapy.
